# Effects of Exercise Training on Molecular Markers of Lipogenesis and Lipid Partitioning in Fructose-Induced Liver Fat Accumulation

**DOI:** 10.1155/2012/181687

**Published:** 2011-08-10

**Authors:** Siham Yasari, Denis Prud'homme, Frédérique Tesson, Marek Jankowski, Jolanta Gutkowska, Emile Levy, Jean-Marc Lavoie

**Affiliations:** ^1^Department of Kinesiology, University of Montreal, C.P. 6128, Succursale Centre-Ville, Montréal , QC, Canada H3C 3J7; ^2^Behavioural and Metabolic Research Unit, Montfort Hospital, University of Ottawa, Ottawa, ON, Canada K1K 0T2; ^3^Laboratory of Genetics of Cardiac Diseases, University of Ottawa Heart Institute, Ottawa, ON, Canada K1Y 4W7; ^4^Research Center, Cardiovascular Biochemistry Laboratory, CHUM-Hôtel-Dieu, University of Montreal, Montreal, QC, Canada H2W 1T8; ^5^Research Center, Sainte-Justine Hospital, University of Montreal, Montreal, QC, Canada H3T 1C5

## Abstract

The present study was designed to investigate the impact of exercise training on lipogenic gene expression in liver and lipid partitioning following the ingestion of a high fructose load. Female rats were exercise-trained for 8 wk or kept sedentary before being submitted to a fasting/refeeding protocol. Rats were further subdivided as follow: rats were fasted for 24 h, refed a standard diet for 24 h, starved for another 24 h, and refed with a standard or a high-fructose diet 24 h before sacrifice. Fructose refeeding was associated with an increase in hepatic lipid content, endocannabinoid receptor 1, sterol regulatory element-binding protein1c, and stearoyl-CoA desaturase1 gene expression in both Sed and TR rats. However, desaturation indexes measured in liver (C16 : 1/C16 : 0 and C18 : 1/C18 : 0) and plasma (C18 : 1/C18 : 0) were higher (*P* < 0.01) in TR than in Sed rats following fructose refeeding. It is concluded that exercise training does not significantly affect fat accumulation and the molecular expression of genes involved in lipogenesis after fasting and fructose refeeding but does modify the partitioning of lipids so as to provide more unsaturated fatty acids in liver without affecting liver fat content.

## 1. Introduction

The recent interest in liver fat metabolism has been spurred by the finding that obesity in Western societies results in an accumulation of hepatic lipids that, in turn, is associated with the deterioration of insulin signalling [1–3]. The clinical relevance of lipid handling by the liver has been enlightened by the finding that, when rats are starved and subsequently refed, liver lipids rapidly accumulate, especially when fed carbohydrates [4]. This phenomenon is closely associated to repeated bouts of weight loss and regain, also known as weight cycling or “yo-yo” dieting [5]. At the gene expression level, a food deprivation/refeeding regimen using high-carbohydrate diets results in the upregulation of several hepatic lipogenic enzymes such as fatty acid synthetase (FAS) [6–8] and stearoyl-CoA desaturase1 (SCD1) [9, 10]. Although no study has reported the effects of fasting/refeeding high-carbohydrate diets on endocannabinoid receptor 1 and 2 (CB1-2), experimental evidence suggests that hepatic CB receptors contribute to the development of diet-induced hepatic steatosis through the induction of the lipogenic transcription factor SREBP1c and its target enzymes (i.e., FAS) [11, 12]. Simple sugars, abundantly found in fruit drinks, sports drinks, and soda, seem to strongly induce obesity and hepatic steatosis in comparison to complex carbohydrates [13–15]. Among simple sugars, fructose is the most potently lipogenic inducer that can increase lipogenesis through activation of SREBP1c dependent and independent mechanisms [14, 16]. 

On the other hand, chronic exercise training affects liver fat metabolism by reducing the accumulation of liver lipids in high-fat fed animals and in humans [17–19]. Although these training adaptations would be compatible with a decrease in long-term fat accumulation, it may represent a disadvantage in an acute situation, such as food deprivation/refeeding a high-fructose diet, in which an increase in hepatic lipogenic activity is necessary to rapidly and adequately buffer the large arrival of substrates. Investigating the role of training on the short-term management of fructose by the liver, in a food-deprived/refed situation, has been limited to the impact of an acute bout of exercise that inhibits key hepatic lipogenic enzymes [7, 8]. Proper handling of substrates by the liver may have subsequent implications on plasma TAG clearance and fat storage, which, in turn, has implications on inflammation and atherosclerosis development [20]. By contrast to training, it has been reported that physical inactivity, known to deteriorate muscle insulin signalling [21], impacts the partitioning of saturated versus unsaturated fatty acids toward storage, resulting in a preferential accumulation of palmitate (C16 : 0) in muscle fat [22]. Physical inactivity also decreases dietary palmitate (C16 : 0) but not oleate (C18 : 1) oxidation, suggesting that the desaturation status of fatty acids is an important factor in determining their fate. 

The first purpose of the present study was, therefore, to determine the effects of an 8-week-exercise training program on HFr refeeding-induced lipogenesis by measuring the expression of the lipogenic genes CB1, CB2, SREBP1c, SCD1, and fatty acid amide hydrolase (FAAH), an endocannabinoid degrading enzyme known to be involved in liver lipid infiltration [11, 23, 24]. It is postulated that under the present fasting/refeeding protocol, TR would be associated with an increased hepatic lipogenic activity. The second objective was to examine the effects of TR on lipid partitioning by measuring fatty acid desaturation index of SCD1 activity in liver and plasma expressed under the ratio of monounsaturated to saturated fatty acids (C16 : 1/C16 : 0 and C18 : 1/C18 : 0) in the context of high-fructose refeeding.

## 2. Materials and Methods

### 2.1. Animal Care

Female Sprague-Dawley rats (Charles River, St-Constant, PQ, Canada), weighing 180–200 g (6 weeks of age), upon their arrival were housed individually and had *ad libitum *access to food and tap water (*n* = 40). Female rats were used in the present study to avoid the decrease in food intake and body weight that was observed in exercise-trained male rats [25, 26]. Their environment was controlled in terms of light (12 : 12-h light-dark cycle starting at 6:00 AM), humidity, and room temperature (20–23°C). This experiment was conducted according to the directives of the Canadian Council on Animal Care after the University of Montreal (Montreal, PQ, Canada) approval.

### 2.2. Exercise Training Protocol

Four days after their arrival, all animals were randomly assigned to a sedentary (Sed) or an exercise-trained group (TR). Exercise training consisted of continuous running on a motor-driven rodent treadmill (Quinton Instruments, Seattle, WA) 5 times/week for 8 weeks. Exercise intensity was progressively increased from 15 min/day at 15 m/min, 0% slope, up to 60 min/day at 26 m/min, 10% slope, for the last 4 weeks of the program. Based on previous measurements of oxygen consumption during a progressive exercise test in rats, it was estimated that exercise intensity during the last 4 weeks of the training program occurred at ~65% of maximal oxygen consumption [27]. At the end of this 8-week period, animals were sacrificed 36 to 48 h after the last exercise session.

### 2.3. Dietary Treatment Protocol

During the first complete 7 weeks, Sed and TR animals had free access to a standard diet (SD; 12.5% lipid, 63.2% carbohydrate, and 24.3% protein; kcal) consisting of usual pellet rat chow (Agribrands Canada, Woodstock, ON). Toward the end of the 8th week that corresponded to 4 days prior to sacrifice, animals were submitted to two fasting and refeeding cycles [10]. Sed and TR animals fasted for 24 h, refed the SD diet for 24 h, starved for another 24 h, and then refed either the SD or an isoenergetic high-fructose (HFr) diet for 24 h. The HFr diet consisted of 13% lipid, 66.8% carbohydrate mainly fructose, and 20.2% protein (kcal). Details of the diets are presented in Table 1. Body weight and food intake were monitored 3 times/week in all rats during the first 7 weeks and every day during the fasting/refeeding stage.

### 2.4. Blood and Tissue Samplings

All animals were sacrificed between 09:00 and 11:00 AM. Food was removed from the animals' cage at least 3 h before sacrifice. After complete anaesthesia (pentobarbital sodium, 50 mg/kg ip), the abdominal cavity was rapidly opened along the median line of the abdomen. Blood was rapidly (<45 s) drawn from the abdominal vena cava (~4 mL) into syringes pretreated with EDTA (15%). Thereafter, blood was centrifuged (3,000 rpm for 10 min, 4°C). The liver median lobe was freeze-clamped and used for mRNA, Western blot, and lipid analysis. The mesenteric (Mes), urogenital (Ug), and retroperitoneal (Rp) fat deposits were excised and weighed in that order. Mesenteric fat pad consisted of adipose tissue surrounding the gastrointestinal tract from the gastrooesophageal sphincter to the end of the rectum with special care taken in distinguishing and removing pancreatic cells. Urogenital fat pad included adipose tissue surrounding the kidneys, ureters, and bladder in addition to the ovaries, oviducts, and uterus. Retroperitoneal fat pad was taken from the fat deposit behind each kidney along the lumbar muscles. Skeletal muscles (plantaris, soleus, medial, and lateral gastrocnemius) of the right limb were removed and weighed thereafter. All tissue samples were immediately frozen in liquid nitrogen after being weighed. All plasma and tissue samples were stored at −80°C until analyses.

### 2.5. Analytical Procedures

#### 2.5.1. Quantitative Real-Time PCR

Total RNA was extracted from frozen liver tissue with the use of either TRIzol (Invitrogen Canada Inc, Burlington, ON) or Purelink RNA mini Kit (Invitrogen, Carlsbad, CA) according to the manufacturer's protocol. RNA was treated with DNase (Invitrogen) in order to avoid genomic contamination. Treated RNA was reverse-transcribed into cDNA using either the random hexamer primers (Invitrogen) and reverse transcriptase (Invitrogen), or the transcriptor first-strand cDNA synthesis kit Roche Diagnostics, Mannheim, Germany. Subsequently, we added 2 *μ*L cDNA to 18 *μ*L of a reaction mixture containing SYBR Green Supermix from Bio-Rad Laboratories Inc. for SCD1 and SREBP1c and from Roche Diagnostics Manheim, Germany, for CB1, CB2, and FAAH. As for the previous steps, polymerase chain reaction (PCR) was performed in two different PCR machines. An iCycler IQ Real-Time PCR detection system (Bio-Rad Laboratories, Inc., Hercules, CA) was used for SCD1 and SREBP1c, and a Roche LighCycler 480 Instrument (Roche Diagnostics Manheim, Germany) served for CB1, CB2, and FAAH mRNA quantification. All samples were analysed in duplicate. The gene-specific primers were purchased from Invitrogen Life Technologies Inc. and are listed in Table 2. We optimized the PCR reaction protocol according to manufacturer's recommendations. For SCD1 and SREBP1c, the thermal cycling program was 95°C for 2 min, followed by 40 cycles at 95°C for 25 s, at 60°C for 25 s, and at 72°C for 40 s. For the quantification of CB1, CB2, and FAAH mRNA, the following procedure was used. Preincubation lasted 10 min at 95°C, followed by 45 cycles of PCR at 95°C for 15 s, 60°C for 15 s, and 72°C for 15 s. Following PCR, the melting curve was completed to ensure that only one PCR product was amplified per reaction. The relative gene expression was calculated as a function of 2^−ΔΔCt^ and normalized for *β*-actin transcript level. The equation used in the calculation was as follows: fold induction = 2^−[ΔΔCt]^, where Ct = the threshold cycle, (i.e., the cycle number at which the sample's relative fluorescence rises above background fluorescence) and ΔΔCt = [Ct gene of interest (unknown sample) – Ct *β*-actin (unknown sample) – Ct gene of interest (calibrator sample) – Ct *β*-actin (calibrator sample)].

#### 2.5.2. Western Blot Analysis

Liver tissue was prepared by homogenization in RIPA (Radioimmunoprecipitation Assay) buffer (50 nM Tris HCl pH8, 150 nM NaCl, 1% NP-40, 0,5% sodium deoxycholate, 0,1% sodium dodecyl sulphate (SDS)) and 10^−5^ M protease inhibitors (phenylmethyl sulfonyl fluoride (PMSF), EDTA, and Pepstatin A) then centrifuged at 10,000 *g* for 20 min, at 4°C. The supernatants were collected, and protein concentrations were determined using Bradford assay. Then, 30 *μ*g of total protein were applied to each well of 12% SDS polyacrylamide gel and electrophoresed at 4°C for 2 h at 130 V. The resolved protein bands were then transferred onto nitrocellulose membranes (Hybond-C Extra; Amersham Biosciences) at 30 V for 120 min, at room temperature. The blots were blocked overnight at 4°C with blocking buffer (10% nonfat milk in 10 mmol/L Tris, pH 7.5, 100 mmol/L NaCl, 0.1% Tween 20). The blots were incubated for 1 h at room temperature with primary antibody rabbit polyclonal anti-SCD1 (1 : 2000) kindly provided by Dr. Juris Ozols, then with horseradish peroxidase-labeled donkey antirabbit IgG (1 : 10000 dilution, Amersham Biosciences UK) for 2 hours at room temperature as secondary antibody. Protein loading was normalized for *β*-actin (Sigma monoclonal anti-*β*-actin antibody produced in mouse at 1 : 5000 dilution as a primary antibody and horseradish peroxidase-conjugated IgG anti-mouse as secondary antibody). Finally the blots were visualized using the ECL (enhanced chemiluminescent, Amersham RPN 2132) and exposed to autoradiography film (Eugene, St-Laurent, QC). Density of the bands was performed with the use of Photoshop software.

#### 2.5.3. Liver Tissue Preparation for Lipid Analysis Using the Folch Method [28]

Samples of 150–200 mg frozen liver tissue were homogenized in a saline buffer supplemented with 1% antiprotease (PMSF, Pepstatin, leupeptin) and 1% antioxidant (butylated hydroxytoluene, BHT). Lipid extraction from liver tissue was performed overnight at 4°C with the use of a 2 : 1 chloroform-methanol mixture (vol/vol). Then, saline was added and the lower phase was removed into glass vials and dried under a stream of nitrogen [29]. Tubes were immediately stored at −80°C for further fatty acid measurements.

#### 2.5.4. Plasma and Liver Fatty Acid Composition Analysis and SCD1 Desaturation Index

The method used for fatty acid composition analysis was slightly adapted from the technique described by Lepage and Roy [30]. Plasma (100 *μ*L) or prepared liver tissue (described above) was used for this analysis. The samples were dissolved in 2 mL methanol-hexane 4 : 1 (v/v) mixture containing BHT and an internal fatty acid standard (Nonadecanoic acid C19 : 1; NU-Check Prep. Inc., Elysian, MN, USA). Then, 200 *μ*L of acetyl-chloride was added to each tube. Samples were hydrolysed at 100°C for 60 min, followed by the addition of 5 mL of 6% K_2_CO_3_. The upper phase was extracted and then analysed by gas chromatography. As described by Mainieri et al. [29] using the quantitative values of palmitic acid (C16 : 0), palmitoleic acid (C16 : 1), stearic acid (C18 : 0), and oleic acid (C18 : 1), the desaturation index of SCD1 was determined using the product/substrate ratio (C16 : 1/C16 : 0 and C18 : 1/C18 : 0).

#### 2.5.5. Liver TAG

Liver TAG concentration was estimated from glycerol released after ethanolic KOH hydrolysis by a colorimetric method using commercial kits from Sigma (St. Louis, Missouri, USA). Although this method does not discriminate between glycerol from phospholipids or TAG, Frayn and Maycock [31] have shown that omitting removal of phospholipids leads to only a ± 2% error in the determination of muscle TAG. Thus, although a small amount of free glycerol may be produced from hydrolysis of phospholipids, it is considered negligible.

#### 2.5.6. Plasma FFA and TAG

Plasma total free fatty acid (FFA) concentrations were measured by a colorimetric assay with commercially available kits from Roche Diagnostics (Penzberg, Germany). Plasma TAG was measured using a colorimetric method (Sigma; St. Louis, Missouri, USA).

#### 2.5.7. Plasma Glucose, Insulin, and Leptin

Plasma glucose concentrations were determined with the use of a glucose analyser Yellow Springs Instruments 2300 (Yellow Springs, Ohio, USA). Plasma insulin and leptin concentrations were measured with commercially available radioimmunoassay kits (Linco Research, St-Charles, Missouri, USA).

### 2.6. Statistical Analysis

Values are expressed as means ± S.E. Statistical analyses were performed with the use of a two-way ANOVA for nonrepeated measures using training status and diet as main effects. Fisher LSD post hoc test was applied where appropriate, and *P* values < 0.05 were considered significant.

## 3. Results

Feeding rats an HFr diet after starvation failed to induce changes in body weight, abdominal adiposity, muscle weight, circulating glucose, insulin, and leptin, even though it decreased energy intake (*P* < 0.01) (Table 3). Exercise training, however, was associated with a significant increase in energy intake as well as in the sum of 4 muscle weights and with a decrease in intra-abdominal adiposity and in plasma leptin levels under the two diet conditions (*P* < 0.05).

**Figure 1:**
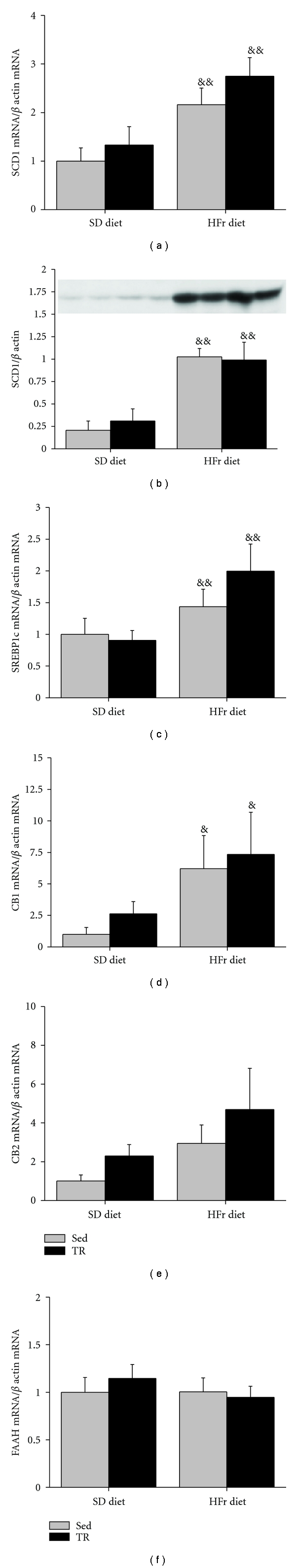
Gene expression of hepatic stearoyl-CoA desaturase1 (SCD1), sterol regulatory element-binding protein1c (SREBP1c), endocannabinoid receptors (CB1 and CB2), fatty acid amide hydrolase (FAAH), and protein content of liver SCD1 relative to *β*-actin in sedentary (Sed) and trained (TR) rats submitted to a fast/refeeding protocol. Animals were fed a standard (SD) diet during 7 wks and thereafter were starved for 24 h and fed with the SD diet for 24 h and starved for another 24 h and then fed with the SD or the high-fructose (HFr) diet 24 h prior to sacrifice at the end of the 8th week. Values are means ± SE with *n* = 10 rats/group except for SCD1 protein level (*n* = 4–6 rats/group). &: significantly different from corresponding values under the SD diet, *P* < 0.05, &&: *P* < 0.01.

HFr compared to SD refeeding resulted in higher (*P* < 0.05) gene expression of SCD1, SREBP1c, and CB1 along with protein content of SCD1 (Figure 1). Training, however, had no effect on the expression of any of these molecular markers.

**Figure 2:**
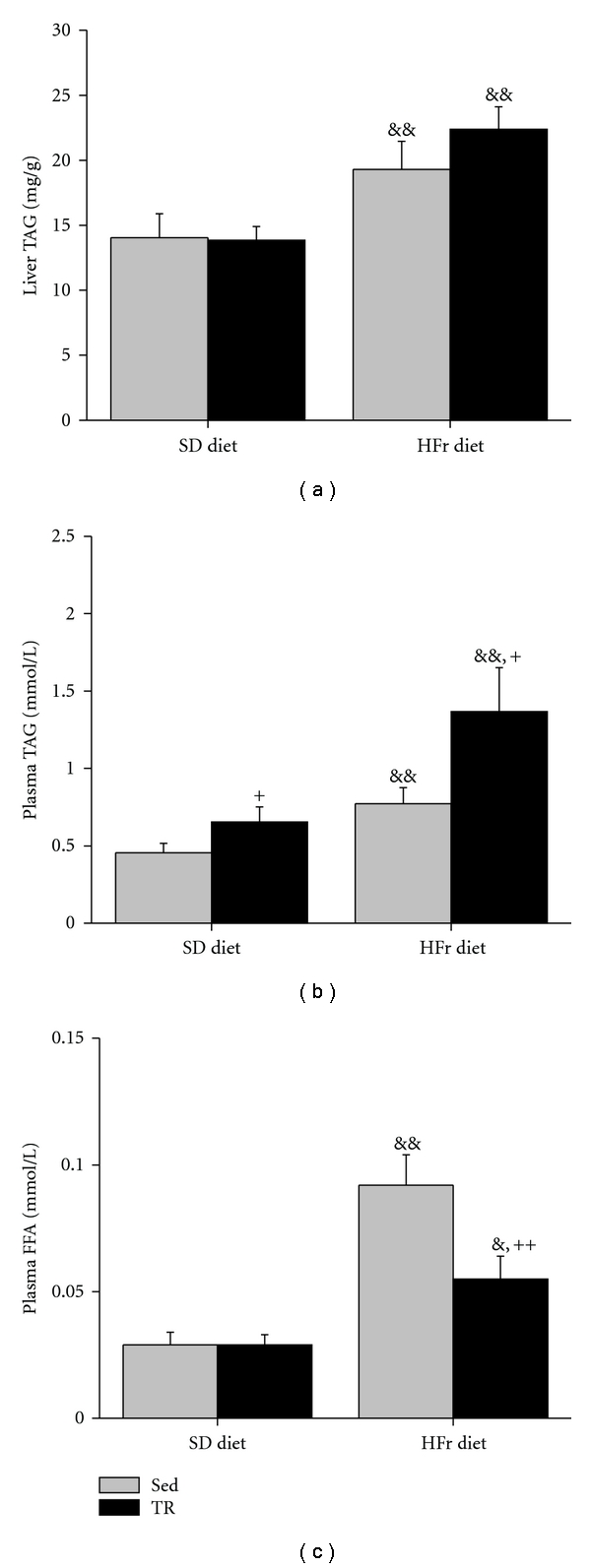
Liver and plasma triacylglycerol (TAG) and plasma free fatty acid (FFA) in sedentary (Sed) and trained (TR) rats submitted to a fast/refeeding protocol. Animals were fed a standard (SD) diet during 7 wks and thereafter were starved for 24 h and fed with the SD diet for 24 h and starved for another 24 h and then fed with the SD or the high-fructose (HFr) diet 24 h prior to sacrifice at the end of the 8th week. Values are means ± SE with *n* = 10 rats/group. &: significantly different from corresponding values under the SD diet, *P* < 0.05, &&: *P* < 0.01. +: significantly different from Sed rats under the same diet condition, *P* < 0.05, ++: *P* < 0.01.

As expected, HFr refeeding resulted in higher liver and plasma TAG as well as plasma FFA levels (Figure 2). Although liver TAG levels were not affected by training, plasma TAG and FFA levels were, respectively, higher (*P* < 0.05) and lower (*P* < 0.01) in TR animals refed with the HFr diet.

**Figure 3:**
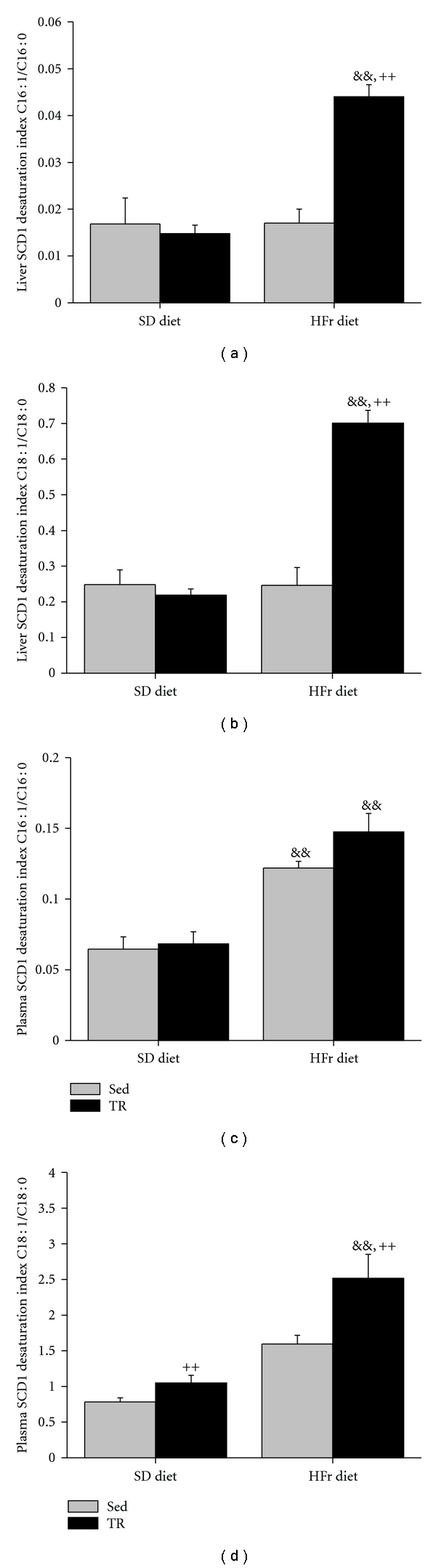
Stearoyl-CoA desaturase1 (SCD1) fatty acid desaturation indexes expressed under the form of the ratio of monounsaturated (C16 : 1, C18 : 1) over saturated (C16 : 0, C18 : 0) fatty acids measured in liver and plasma of sedentary (Sed) and trained (TR) rats submitted to a fast/refeeding protocol. Animals were fed a standard (SD) diet during 7 wks and thereafter were starved for 24 h and fed with the SD diet for 24 h and starved for another 24 h and then fed with the SD or the high-fructose (HFr) diet 24 h prior to sacrifice at the end of the 8th week. Values are means ± SE, *n* = 10 rats/group for plasma and *n* = 5 rats/group for liver desaturation indexes. &&: significantly different from corresponding values under the SD diet, *P* < 0.01. ++: significantly different from Sed rats under the same diet condition, *P* < 0.01.

SCD1 fatty acid desaturation indexes (C16 : 1/C16 : 0 and C18 : 1/C18 : 0) in the liver were significantly (*P* < 0.01) increased exclusively in TR animals refed the HFr diet as a result of a synergetic effect of TR and HFr refeeding (Figures 3(a) and 3(b)). In plasma, HFr refeeding induced an increase in C16 : 1/C16 : 0 in both Sed and TR rats and an increase in C18 : 1/C18 : 0 only in TR rats (Figures 3(c) and 3(d)).

## 4. Discussion

In an attempt to further understand the role played by regular exercise training on liver fat metabolism and accumulation, we designed the present study to determine if an exercise training program may affect the acute handling of a fructose load by the liver. Using a fasting/HFr refeeding approach, we found that gene expression of hepatic molecular markers of the lipogenesis pathway and as well liver fat accumulation were highly but similarly increased by the HFr refeeding in Sed as well as in TR rats. To go one step further, we measured the ratio of monounsaturated to saturated fatty acids, which is used as a surrogate marker for SCD1 activity in liver, thus providing a physiological assessment of the fructose disposal by the liver and fatty acid partitioning [14, 32]. We report for the first time that, following the ingestion of a fructose load, the SCD1desaturation indexes were higher in liver (C16 : 1/C16 : 0 and C18 : 1/C18 : 0) and in plasma (C18 : 1/C18 : 0) of TR compared to Sed animals. This finding indicates that training affects the acute handling of a fructose load by providing a higher rate of conversion of *de novo* synthesized saturated fatty acids to the monounsaturated form.

The HFr refeeding in Sed animals did not affect the fatty acids desaturation indexes measured in the liver (Figures 3(a) and 3(b)). This indicates that the HFr diet did not result in a change in the ratio of saturated versus monounsaturated fatty acids accumulated in the liver. However, the C16 : 1/C16 : 0 desaturation index of Sed rats refed with the HFr was highly increased in plasma, suggesting an increased liver exportation of the fatty acids under the desaturated form. Several studies have demonstrated that fatty acids originating from different sources appear to be managed differently in the liver with regard to their use for storage or secretion [33, 34]. More specifically, it seems that newly synthesized fatty acids are channelled preferentially into very-low-density lipoproteins for exportation [35]. Plasma TAG and FFA plasma levels were accordingly increased by the HFr refeeding in Sed rats. However a somewhat different picture is found in TR rats. First, liver SCD-1 desaturation indexes were highly increased following training without any effects on liver TAG levels. This indicates that although the same quantity of fat was accumulating in liver of Sed and TR animals after the HFr refeeding, more of the fatty acids stored in liver of TR rats were under the desaturated form. This as such provides new evidence that training affect, the acute handling of a fructose load. It might be argued that the increased desaturation ratio in liver of TR rats might be due to a decreased exportation of desaturated fatty acids. This possibility, however, is not supported by the finding that the C16 : 1/C16 : 0 desaturation ratio in plasma was similar between Sed and TR rats and the C18 : 1/C18 : 0 ratio was higher in TR than in Sed animals. The present data, therefore, support the interpretation that exercise training may influence the acute management of a substrate load such as fructose by changing the partitioning of newly synthesized fatty acids. Supporting this view is the recent report that physical inactivity favours the accumulation of palmitate (C16 : 0) in muscle fat and decreases dietary palmitate but not oleate (C18 : 1) oxidation thus leading to the deterioration of insulin signalling [22]. 

The higher levels of fatty acids under the desaturated form in liver of TR rats refed with the HFr load may present some advantages. Recently, Li et al. [36] reported that SCD1 plays a key role in prevention of steatohepatitis by partitioning excess lipid into monounsaturated fatty acids (MUFAs) that can be safely stored. This indicates that training favouring the formation of fatty acids that are mostly unsaturated in liver could represent a molecular mechanism of exercise-training-induced metabolic protection in liver. Newly synthesized MUFAs C16 : 1 and C18 : 1 are the most abundant fatty acids founds in TAG molecules [37]. In this regard, exercise training might favour the conversion of the *de novo* synthesized fatty acids that are cytotoxic molecules into a form more easily transferrable to TAG, known to be biologically inert molecules [38, 39]. 

Exercise training did not, under the present acute dietary manipulations, result in lower levels of liver fat accumulation. The effects of exercise training in reducing liver fat accumulation under long-term high-fat diets have been reported in several studies [17, 40]. The absence of effects of exercise training on liver fat accumulation in the present study is most likely due to the powerful effect of the HFr load on lipogenesis. HFr refeeding, whether in Sed or TR rats, was associated with an increase in hepatic gene expression of SCD1, SREBP1c, and the endocannabinoids CB1 receptors. It has been reported that the activation of CB1 receptors in liver stimulates *de novo* lipogenesis through induction of SREBP1c and its target enzymes [12]. Recent data in liver-specific CB1 knockout mice indicate that hepatic CB1 receptors are required for the development of diet-induced steatosis by increasing *de novo* lipogenesis and inhibiting fatty acid oxidation [11]. In agreement with this statement, our data show, for the first time, that gene expression of CB1, and to a lesser extent CB2, was increased in liver of fasted and fructose refed animals in comparison to those refed the SD diet. Additionally, it has been reported that lipid accumulation in liver may also occur by reduced expression and/or activity of fatty acid amide hydrolase (FAAH), one of the endocannabinoid degrading enzymes [24]. The present results, however, do not indicate any effects of the high-fructose diet on the gene expression of the FAAH enzyme. 

The fact that SCD1 gene expression and protein content in liver were not changed in TR compared to Sed rats under the HFr refeeding might at first glance contradict the finding of higher SCD1 desaturation indexes measured in the same rats. As stated above, the SCD1 index reflects the activity of SCD1. It is possible that since the HFr load highly stimulated the lipogenesis pathway that the discrimination between the TR and the Sed state might be possible only at the activity level. 

An intriguing observation that needs to be addressed is the fact that several hours after the HFr refeeding, TR rats had higher plasma TAG and lower plasma FFA levels than their Sed counterparts. The present study provides no information on VLDL secretion rate. However, since liver TAG levels were similar in TR and Sed rats, it is unlikely that a difference in VLDL synthesis and/or secretion rate is responsible for the higher plasma TAG levels found in TR animals. In search of a different explanation, we must first acknowledge the fact that energy intake was ~30% higher in TR than in Sed rats refed the HFr diet. On a speculative basis, it is possible that the lower plasma TAG levels found in Sed compared to TR rats might be linked to a greater ability of Sed rats to store fat in peripheral adipose tissue under the present acute nutritional manipulations. Although the inter-diet comparisons did not reach the statistically significant level, the HFr compared to the SD refeeding seems to have resulted in higher intra-abdominal fat accumulation in Sed (from ~16 to 21 g) than TR rats (from 16 to 15.8 g; Table 3). In connection with this, higher plasma FFA levels measured in Sed compared to TR rats refed the HFr could result from a higher rate of basal lipolysis due to the higher adipocyte fat accumulation [41].

In summary, exercise training is associated with higher liver (C16 : 1/C16 : 0 and C18 : 1/C18 : 0) and plasma (C18 : 1/C18 : 0) SCD1 desaturation indexes in rats submitted to a 2-day fast/refeeding protocol using a high-fructose diet in the second day. Gene expression of lipogenic molecular markers including hepatic endocannabinoid receptors, transcription factor SREBP1c, and SCD1 protein content was increased to a similar in extent in Sed and TR rats refed with the HFr diet. It is concluded that the pattern of management of the HFr load in a fast/refed protocol is modified in exercise trained animals so as to provide more fatty acids under the unsaturated form in liver and plasma. These data support the contention that exercise training positively modifies the handling of an acute substrate load.

## Figures and Tables

**Table 1:** Composition of the diets.

Ingredients	Amount (Kcal %)	
	SD^¥^	HFr*
Fat	**12. 5%**	**13%**
Saturated	30 %	42 %
Unsaturated	70 %	58 %
Carbohydrate	**63.2 %**	**66.8 %**
Protein	**24.3 %**	**20.2 %**

Gross energy Kcal/g	3.5	3.6

SD: standard diet, HFr: high-fructose diet.

^¥^Standard laboratory rat chow (5075, Charles River). The main carbohydrate is starch (65 % of total carbohydrates).

*High-fructose diet (TD 89247, Harlan Teklad). The main carbohydrate is fructose (99.23 % of total carbohydrates).

**Table 2:** Real-time PCR primer sequences.

Gene	Sense (5′-3′)	Antisense (5′-3′)	Accession no.
CB1	AAGGACCTGAGACATGCTTTCCGA	TCGCGATCTTAACGGTGCTCTTGA	X55812
CB2	GGGGTGGACTTGTTGTCCTA	ACATGTTGGTGTGCTTTCCA	NM_020543
FAAH	ACGATGCCCAGATGGAACTCTACA	GCATGAACCTCAGACACAGCTCTT	U72497
SCD1	CCTTAACCCTGAGATCCCGTAGA	AGCCCATAAAAGATTTCTGCAAA	J02585
SREBP1c	ACGACGGAGCCATGGATTGCAC	CCGGAAGGCAGGCTTGAGTACC	L16995
*β*-actin	CATGAAGATCAAGATCATTGCTCCT	CTGCTTGCTGATCCACATCTG	V01217

CB1: endocannabinoid receptor1.

CB2: endocannabinoid receptor2.

FAAH: fatty acid amide hydrolase.

SCD1: stearoyl-CoA desaturase1.

SREBP1c: sterol regulatory element-binding protein1c.

**Table 3:** Body weight, energy intake, sum of intra-abdominal fat pad weights (mesenteric, urogenital, and retroperitoneal), sum of 4 muscle weights (soleus, plantaris, medial, and lateral gastrocnemius), plasma glucose, insulin, and leptin in sedentary (Sed) and trained (TR) rats submitted to a fasting/refeeding protocol. Animals were fed a standard (SD) diet during 7 wks and thereafter were starved for 24 h and fed a SD diet for 24 h and starved for another 24 h and then fed the SD or the high-fructose (HFr) diet 24 h prior to sacrifice at the end of the 8th week.

Diets	SD diet		HFr diet	
	Sed	TR	Sed	TR
Body weight (g)	281 ± 8	292 ± 8	287 ± 9	291 ± 7
Energy intake (kcal/last 24 h)	84.7 ± 2.9	88.2 ± 4.2^+^	57.9 ± 4.5^&&^	74.9 ± 4.9^&&,+^
Sum of intra-abdominal fat pad weights (g)	16.12 ± 1.9	15.2 ± 1.6^+^ (*P* = 0.058)	21.3 ± 2.37	14.8 ± 1.58^+^ (*P* = 0.058)
Sum of 4 muscle weights (g)	1.96 ± 0.05	2.08 ± 0.05^+^	1.97 ± 0.04	2.07 ± 0.05^+^
Plasma glucose (mmol/L)	7.7 ± 0.3	8.1 ± 0.3	8.1 ± 0.4	7.3 ± 0.2
Plasma insulin (pM)	456 ± 67	573 ± 66	437 ± 65	447 ± 59
Plasma leptin (ng/mL)	2.62 ± 0.4	2.26 ± 0.2^+^	3 ± 0.4	2.02 ± 0.2^+^

Values are means ± SE, *n* = 10 rats/group.

^&&^Significantly different from corresponding values under the SD diet, *P* < 0.01. ^+^Significantly different from Sed rats under the same diet condition, *P* < 0.05.
